# Supplementary material for: Single-cell transcriptome analysis of lineage diversity in high-grade glioma
Source: Genome Med. 2018 Jul 24;10:57. doi: 10.1186/s13073-018-0567-9 (PMC6058390; doi:10.1186/s13073-018-0567-9)
Supplement: Supplementary file 1 — Supplementary figures and tables. (DOCX 17628 kb) [file 13073_2018_567_MOESM1_ESM.docx]

**
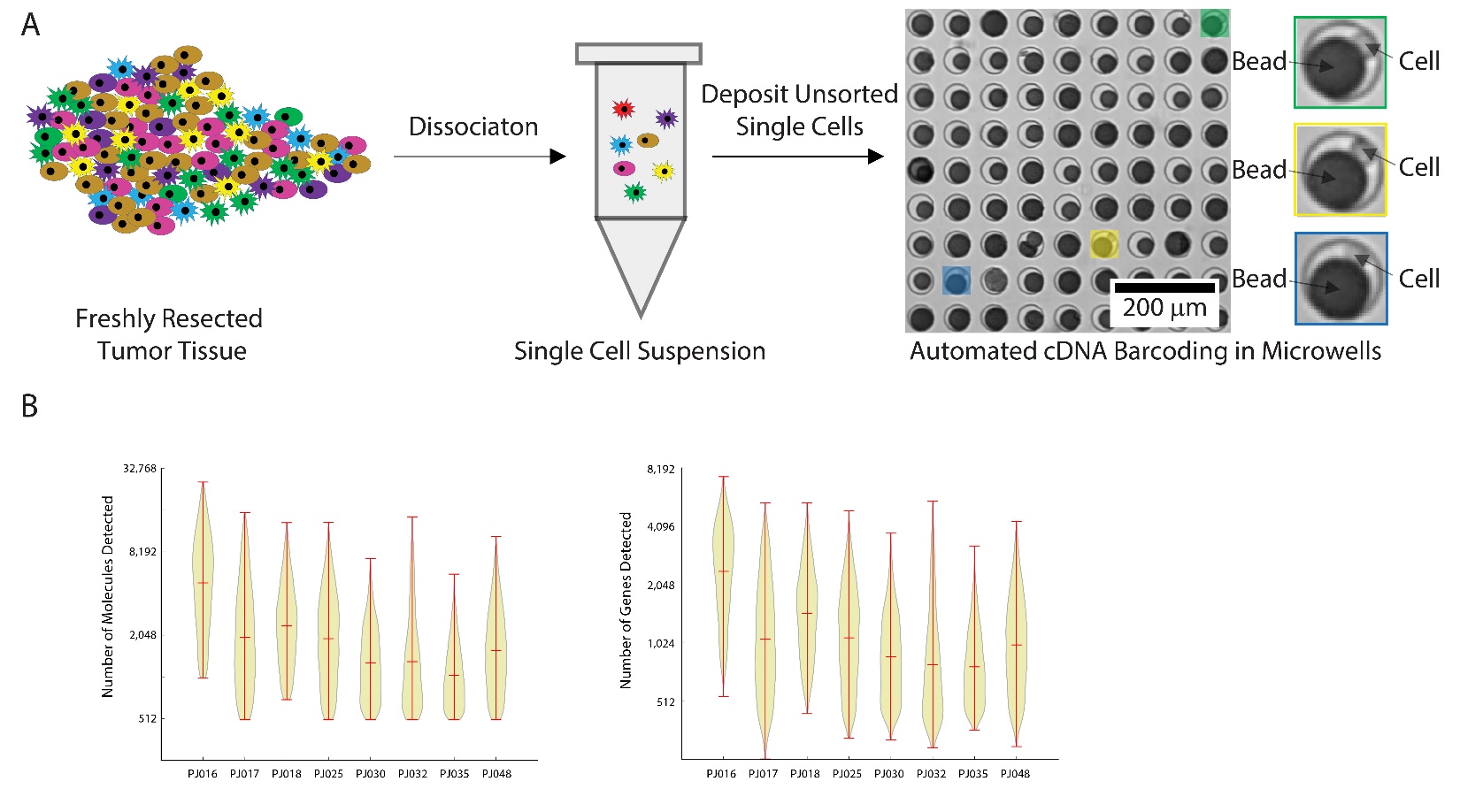
**

**Figure S1.** A) Schematic of the experimental workflow for large-scale scRNA-Seq of unsorted single cell suspensions dissociated from fresh, HGG surgical specimens. B) Violin plots showing the numbers of molecules (left) and genes (right) detected per cell in each HGG sample.


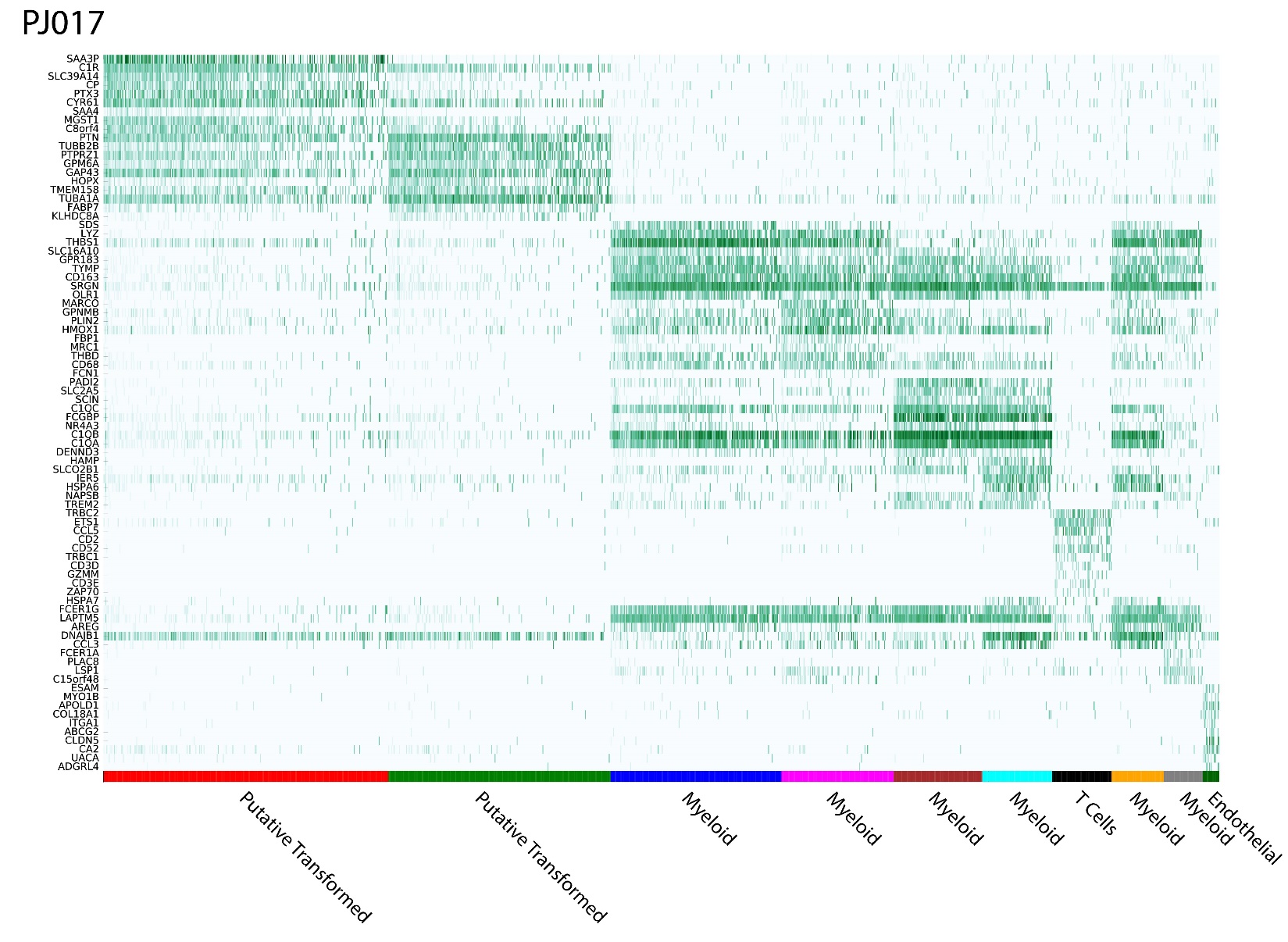


**Figure S2.** Heatmap showing the top 10 most specific genes for each Phenograph cluster from the HGG PJ017 and the corresponding cell type.

**
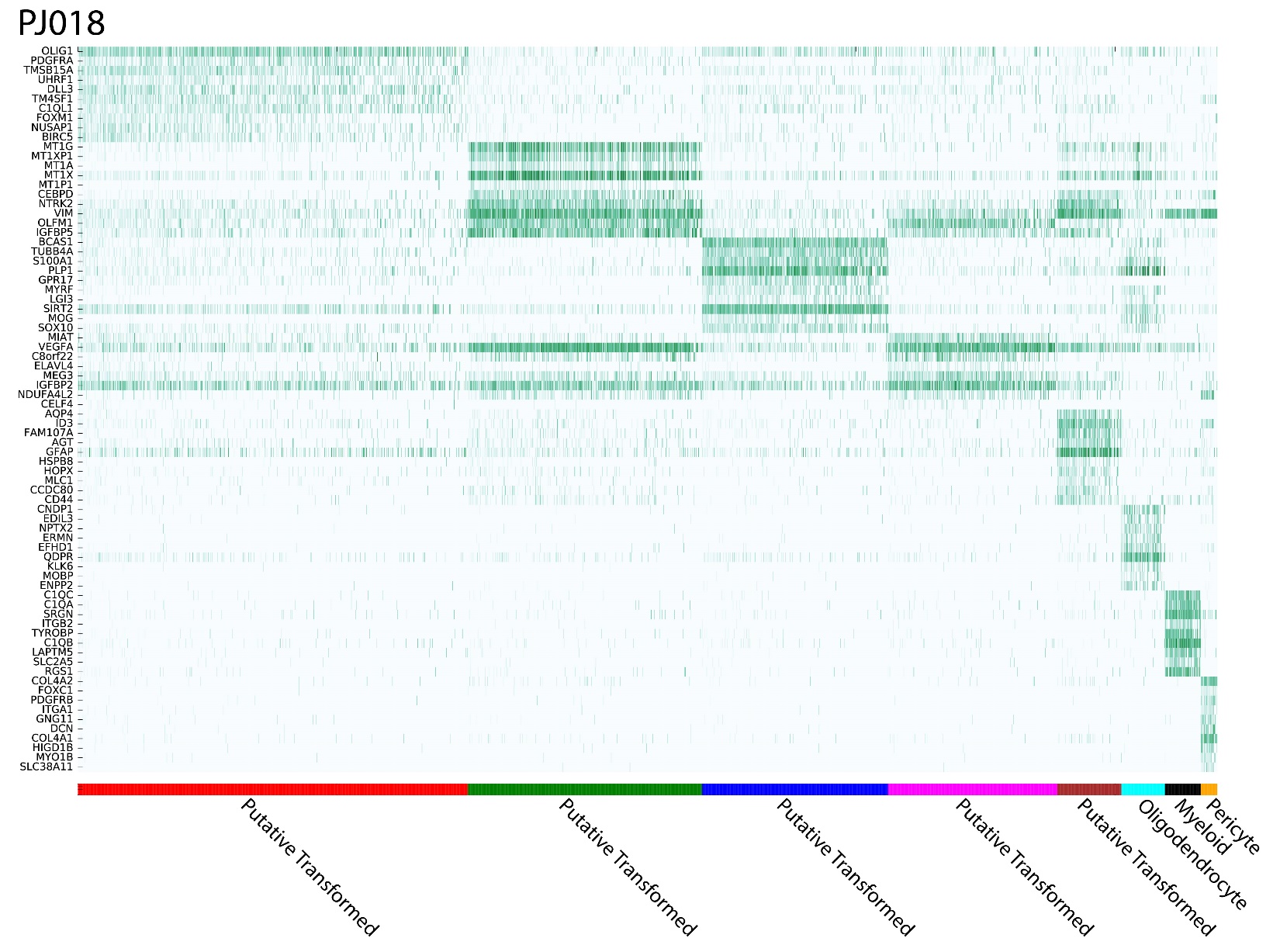
**

**Figure S3.** Heatmap showing the top 10 most specific genes for each Phenograph cluster from the HGG PJ018 and the corresponding cell type.

**
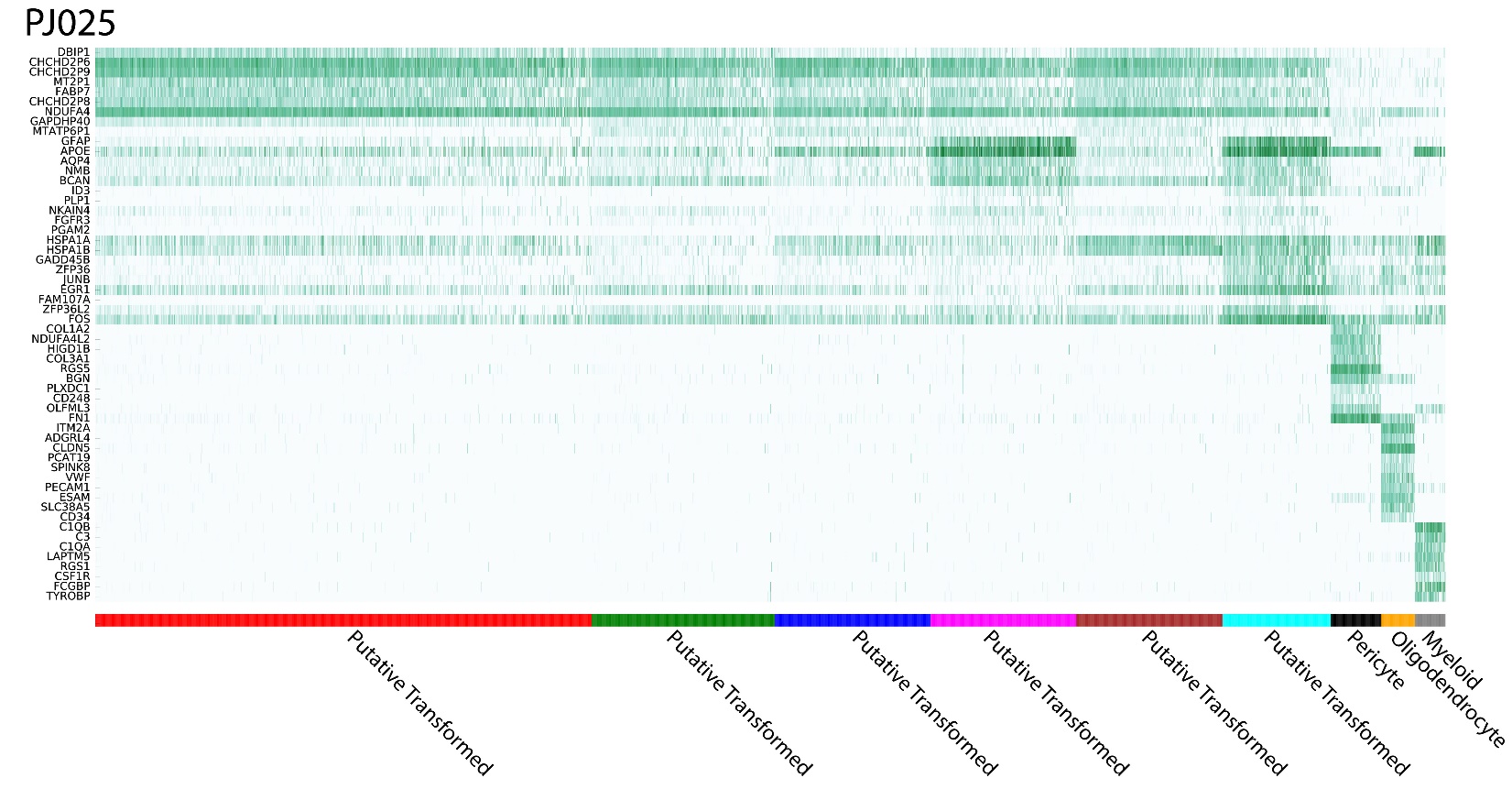
 Figure S4.** Heatmap showing the top 10 most specific genes for each Phenograph cluster from the HGG PJ025 and the corresponding cell type.

**
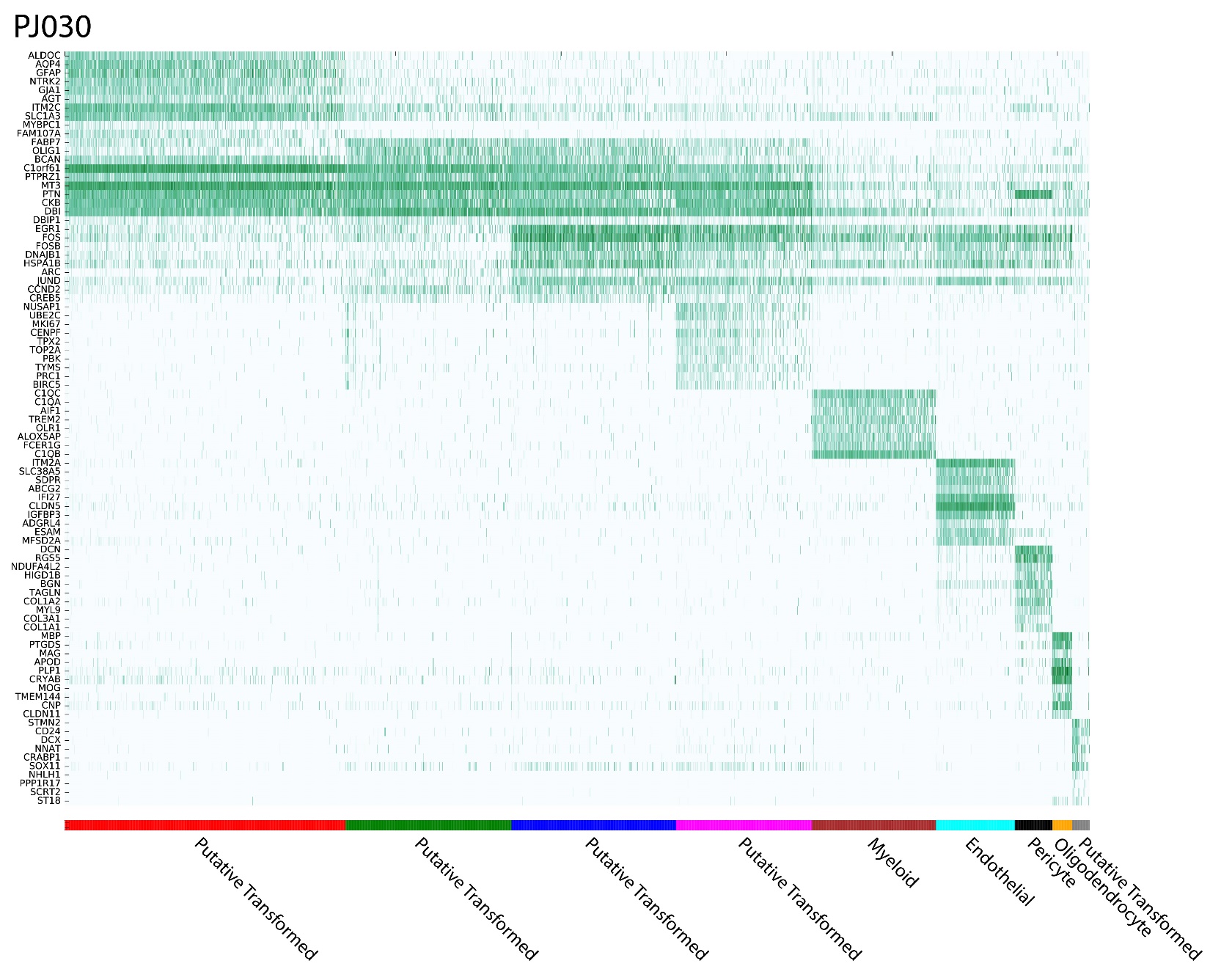
**

**Figure S5.** Heatmap showing the top 10 most specific genes for each Phenograph cluster from the HGG PJ030 and the corresponding cell type.

**
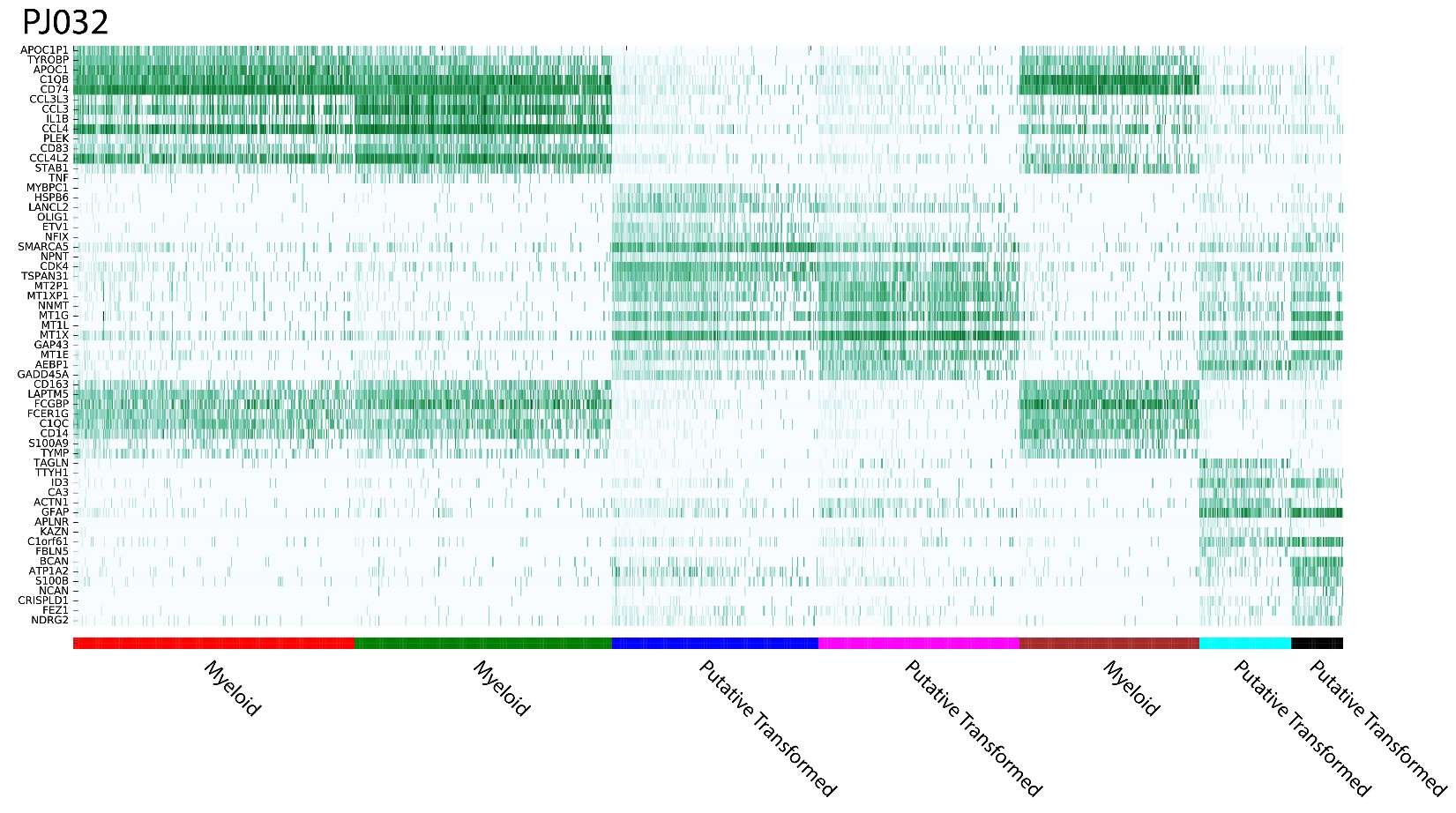
**

**Figure S6.** Heatmap showing the top 10 most specific genes for each Phenograph cluster from the HGG PJ032 and the corresponding cell type.

**
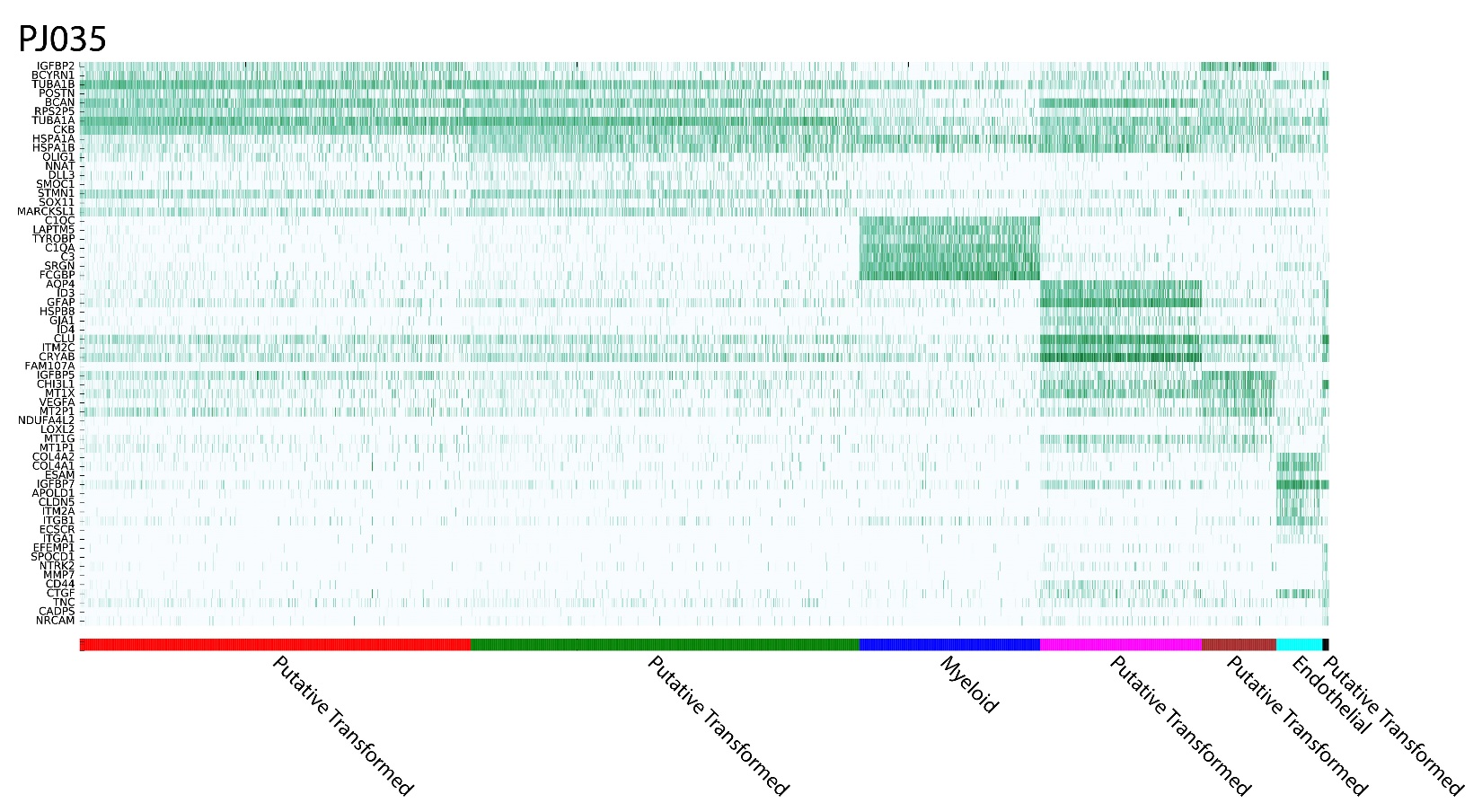
**

**Figure S7.** Heatmap showing the top 10 most specific genes for each Phenograph cluster from the HGG PJ035 and the corresponding cell type.

**
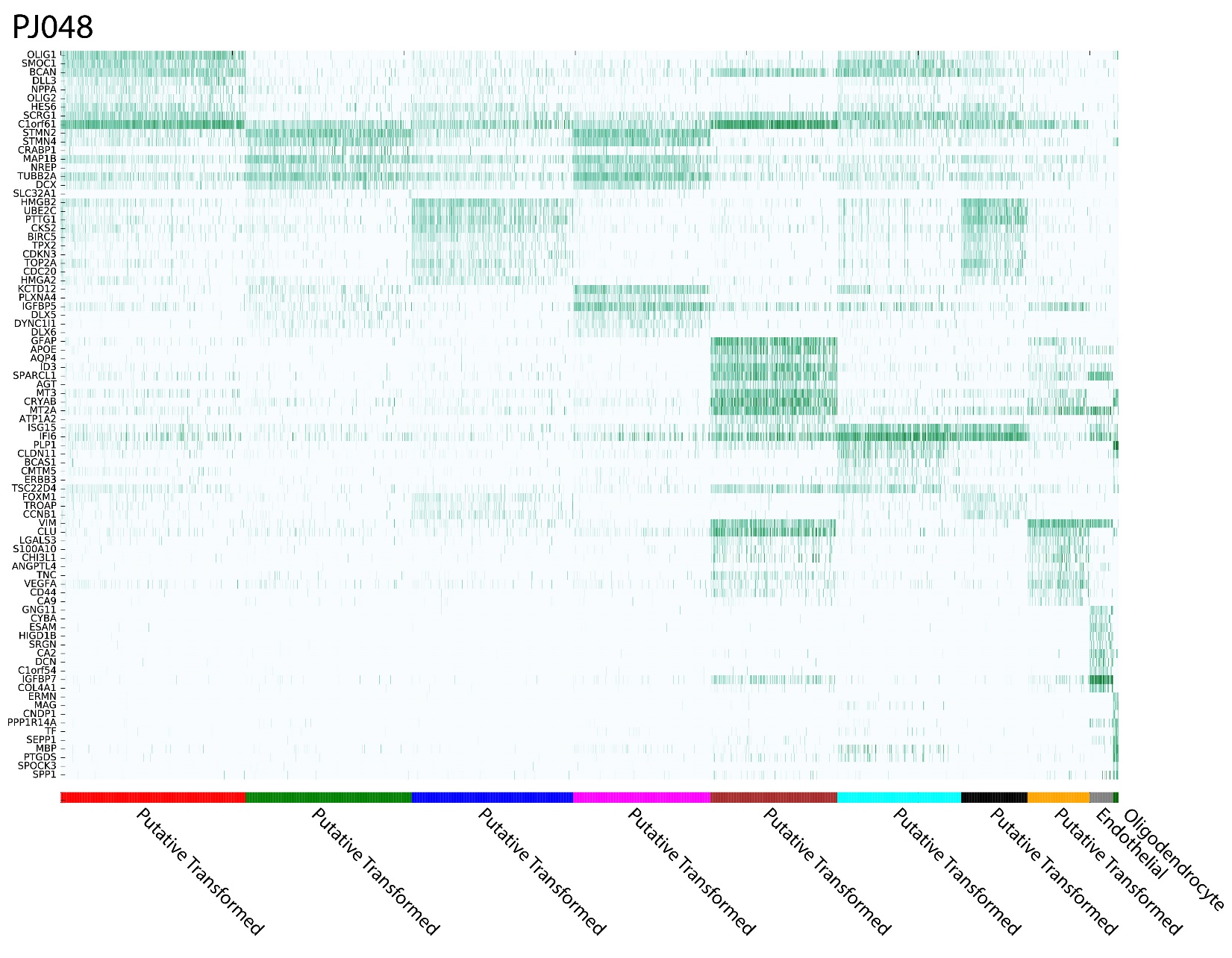
**

**Figure S8.** Heatmap showing the top 10 most specific genes for each Phenograph cluster from the HGG PJ048 and the corresponding cell type.

**
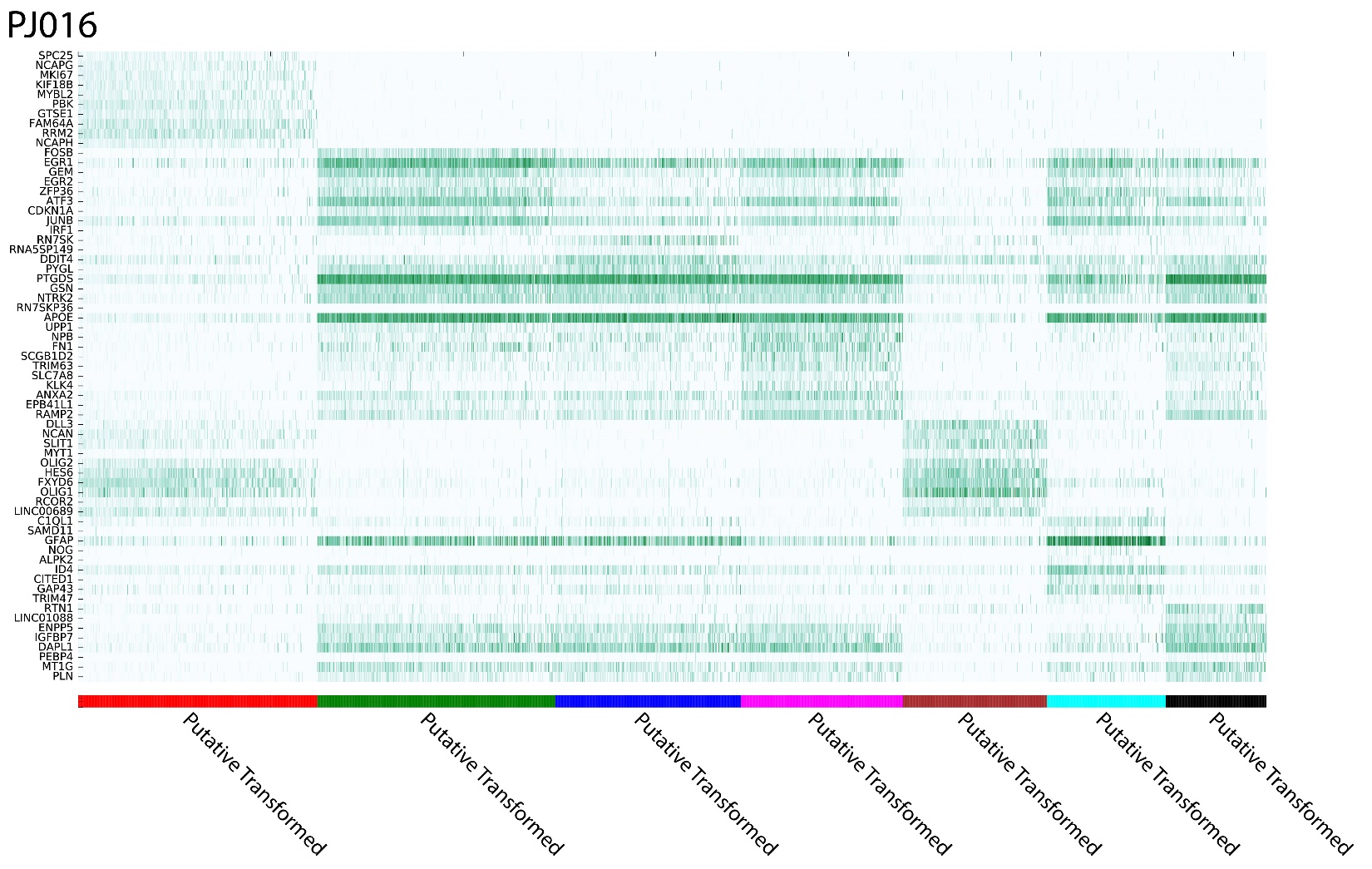
**

**Figure S9.** Heatmap showing the top 10 most specific genes for each Phenograph cluster from the HGG PJ016 and the corresponding cell type.

**
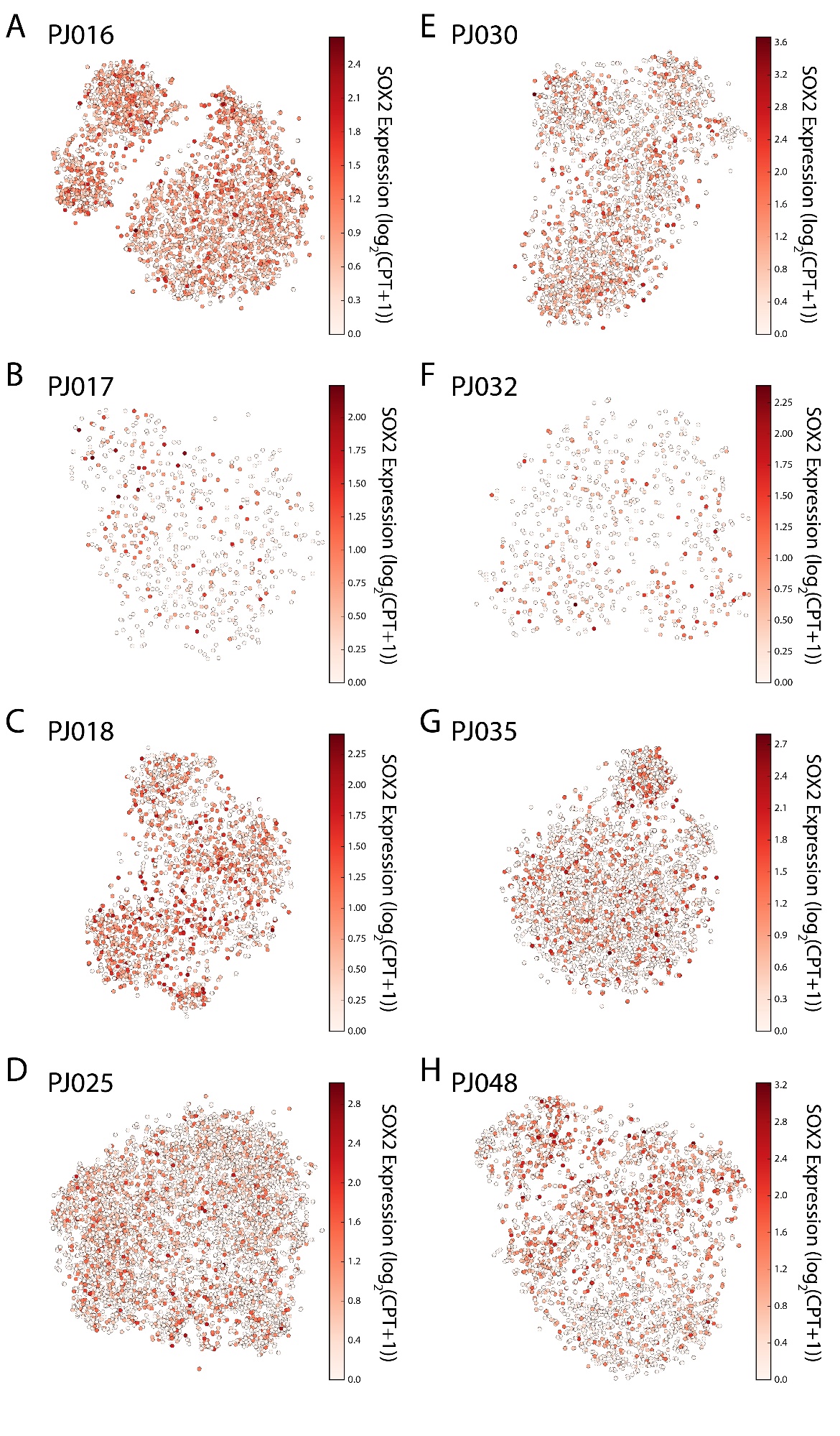
**

**Figure S10.** tSNE plots colored by expression of *SOX2* in the transformed cells from each tumor. Expression is given in log-transformed counts per thousand counts.

**
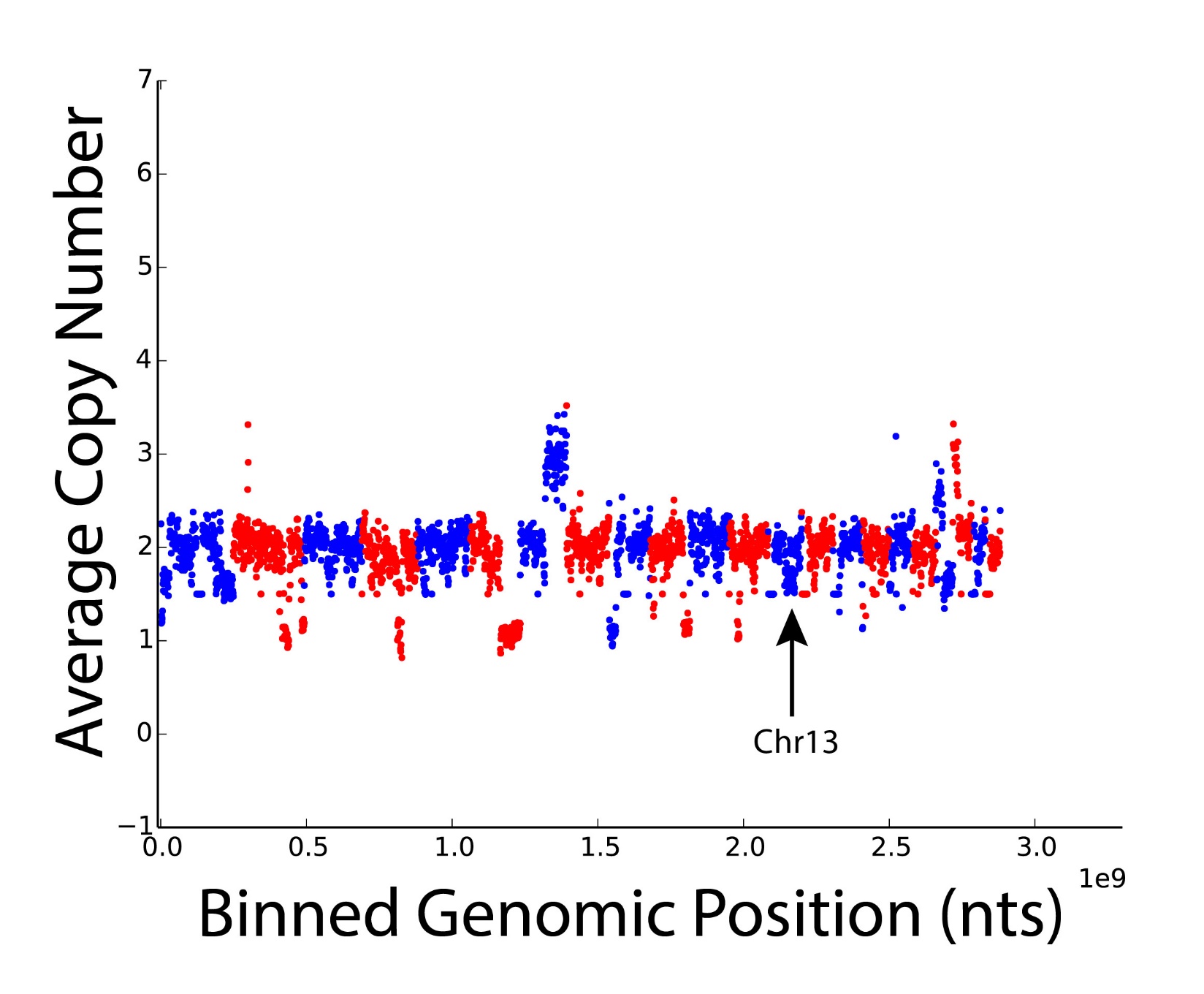
**

**Figure S11.** Genome-wide average copy number from low-pass bulk WGS in PJ016 tumor tissue binned to 1M nucleotides. We see a clear loss of a region of Chr13 which, although unclear from the bulk WGS heatmap in **Figure 1E** where we average over the entire chromosome, is pronounced in the heatmap generated from scRNA-Seq data in **Figure 1E**.

**Figure S12.** Transformed cell fraction based on scRNA-Seq (using the analysis in **Figure 1**) vs. SOX2 labeling index from immunohistochemical staining of the corresponding tumor tissue for six patients. The two variables are highly correlated (r = 0.98, p = 0.001).


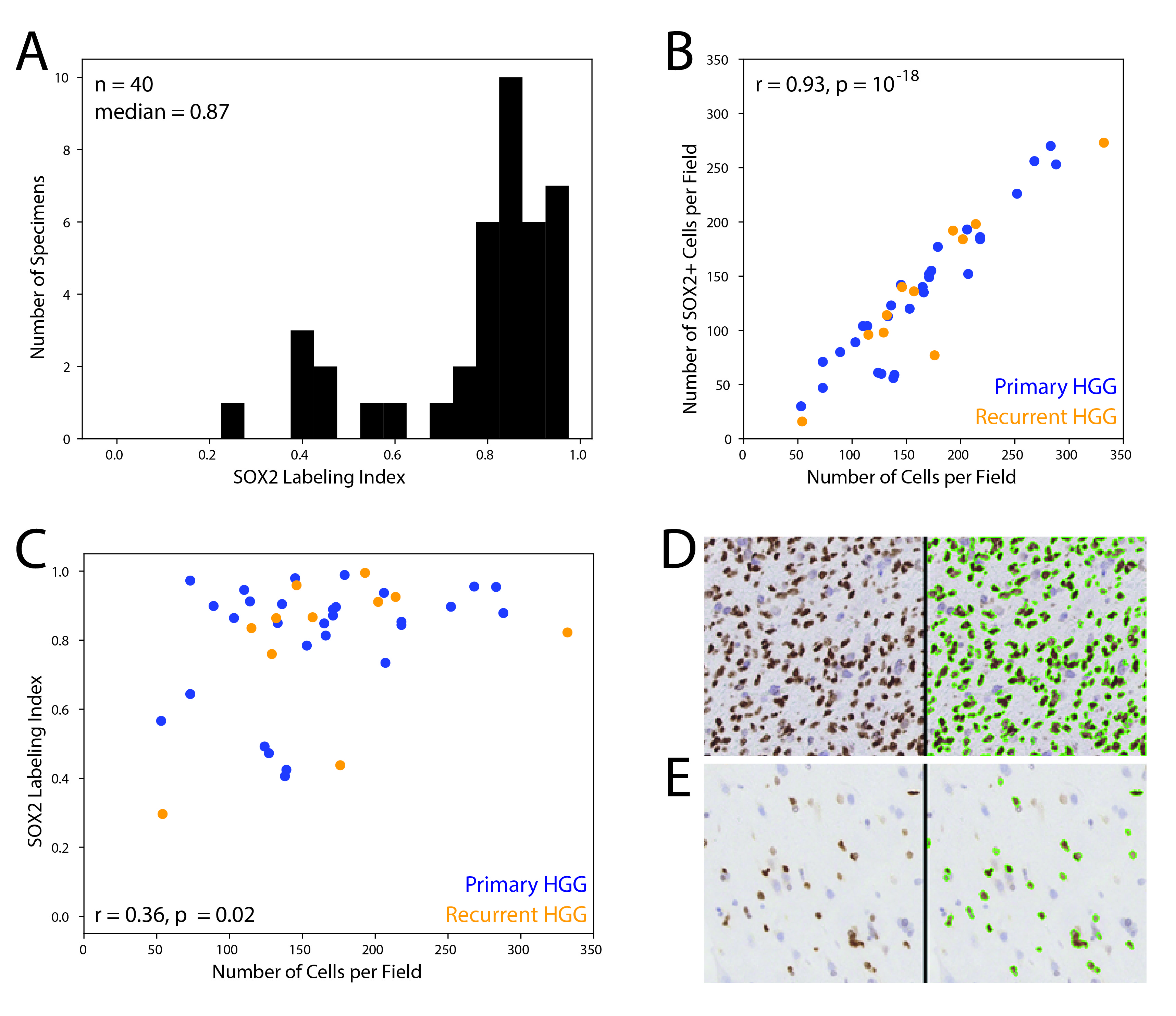


**Figure S13.** Validation of SOX2 expression in a cohort of 40 HGG specimens. A) SOX2 labeling index distribution showing a median labeling index of 0.87 (fraction of cells expressing SOX2). B) Scatter plot showing the correlation between SOX2 labeling density (number of SOX2+ cells per field-of-view) and overall cell density (number of cells per field-of-view) in both primary and recurrent HGG. C) Scatter plot showing the correlation between SOX2 labeling index and overall cell density in both primary and recurrent HGG. D) Representative high-cellularity field-of-view showing SOX2 immunohistochemical staining (brown) and hematoxylin counter-stained nuclei (blue). The left panel shows the original image and the right panel indicates the SOX2+ nuclei identified by the computer program with a green outline. E) Same as D) but for a low cellularity field-of-view.


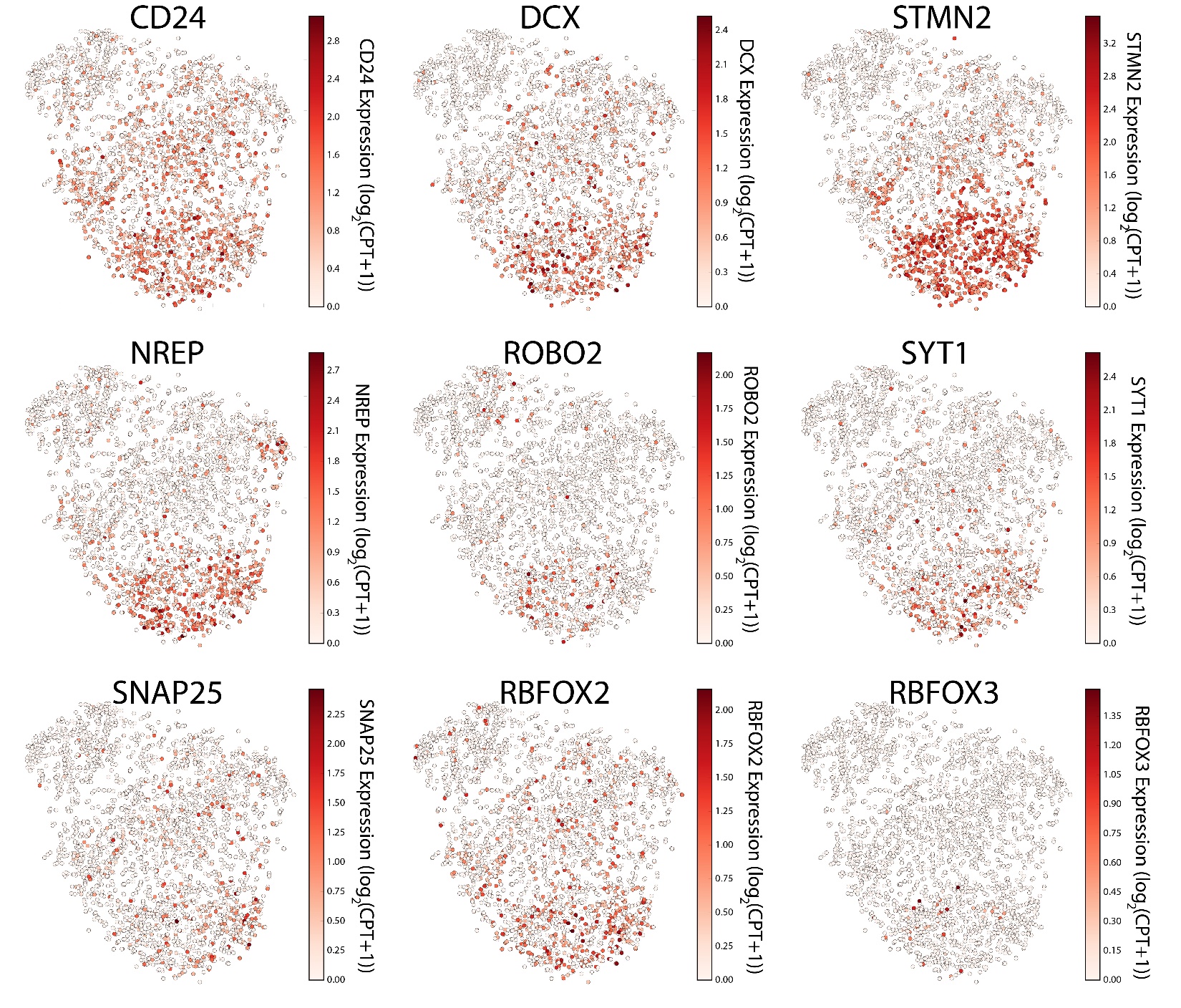


**Figure S14.** tSNE plots for the transformed cells in PJ048 showing expression of genes involved in neuronal differentiation, maturation, and function concentrated in a subpopulation of cells near the lower portion of the projection.


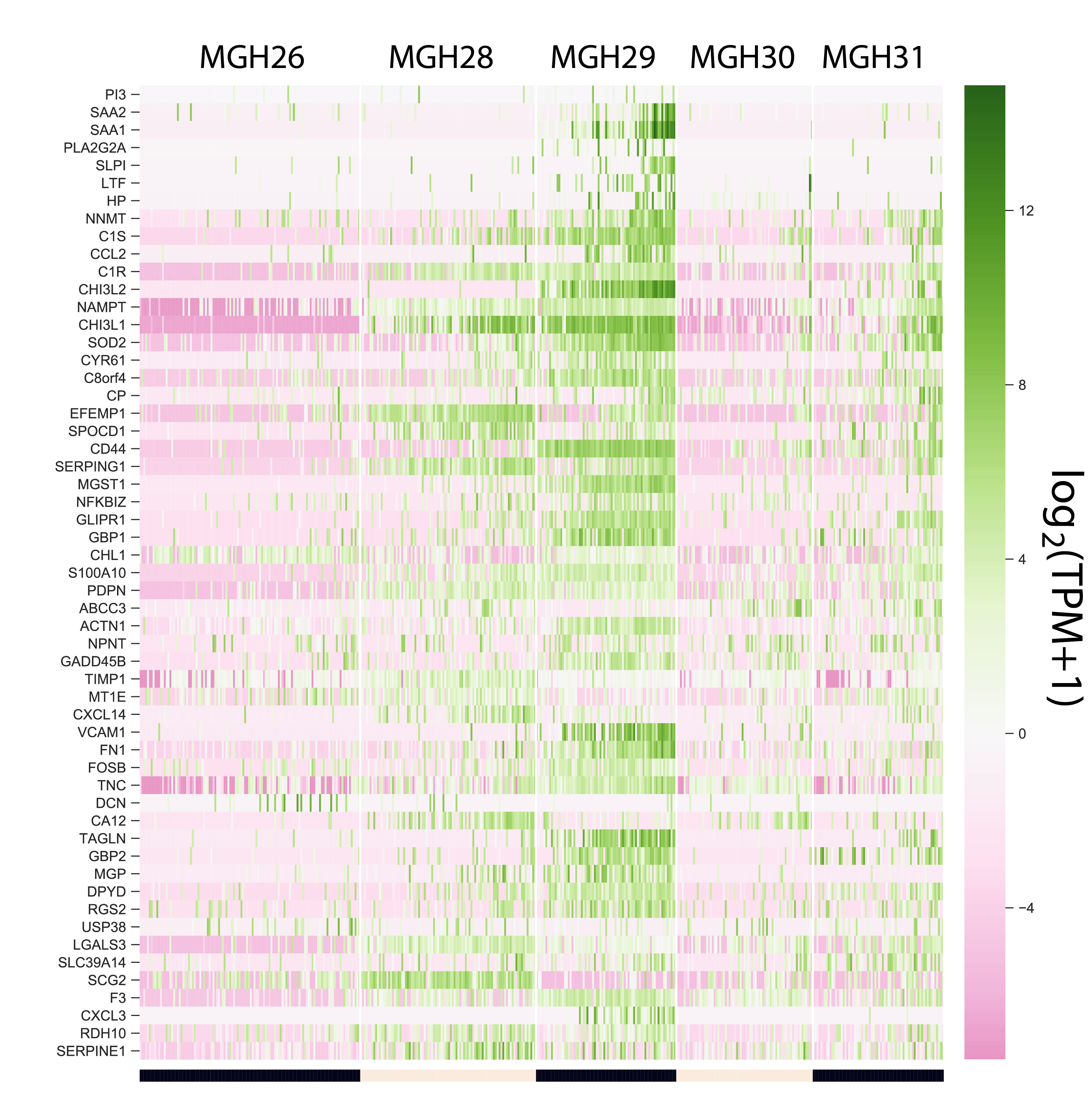


**Figure S15**. Relative expression of PJ017 and PJ032 tumor cell specific genes (listed in Supplementary Table S3) in an independent cohort of GBM patient samples sequenced with single-cell RNA-Seq from Patel *et al*. This set of markers is significantly enriched in immune/inflammatory gene signatures. The transformed GBM cells in MGH29 show markedly higher expression of this gene signature, demonstrating the relevance of our finding in an independent cohort from an earlier study.


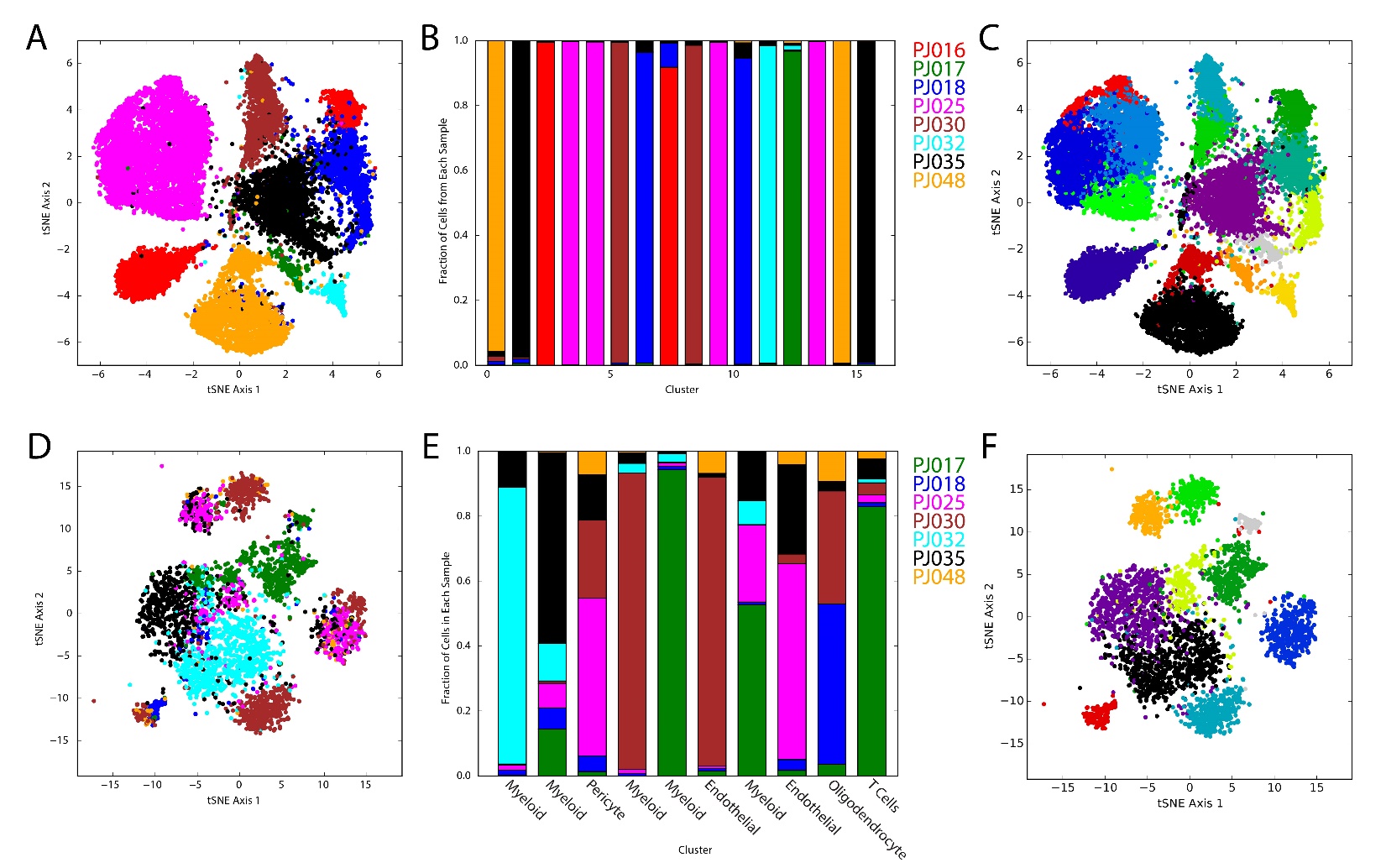


**Figure S16.** A) tSNE projection of the combined transformed cells from all eight tumors colored by sample. B) Sample composition of the 16 Phenograph clusters that result from combining the transformed cells from all eight tumors. The transformed cells cluster mainly by patient. C) Same as A) but colored by Phenograph cluster. D) tSNE projection of the combined untransformed cells from the seven tumors in which untransformed cells were detected. E) Sample composition of the 10 Phenograph clusters that result from combining the untransformed cells, labeled by cell type. Many clusters have a substantial number of cells from multiple tumors, suggesting that the untransformed cells do not cluster exclusively by patient. The myeloid cells are a notable exception to this along with one of the two endothelial clusters (very few T Cells were detected and mainly originated from a single tumor). F) Same as D) but colored by Phenograph cluster.


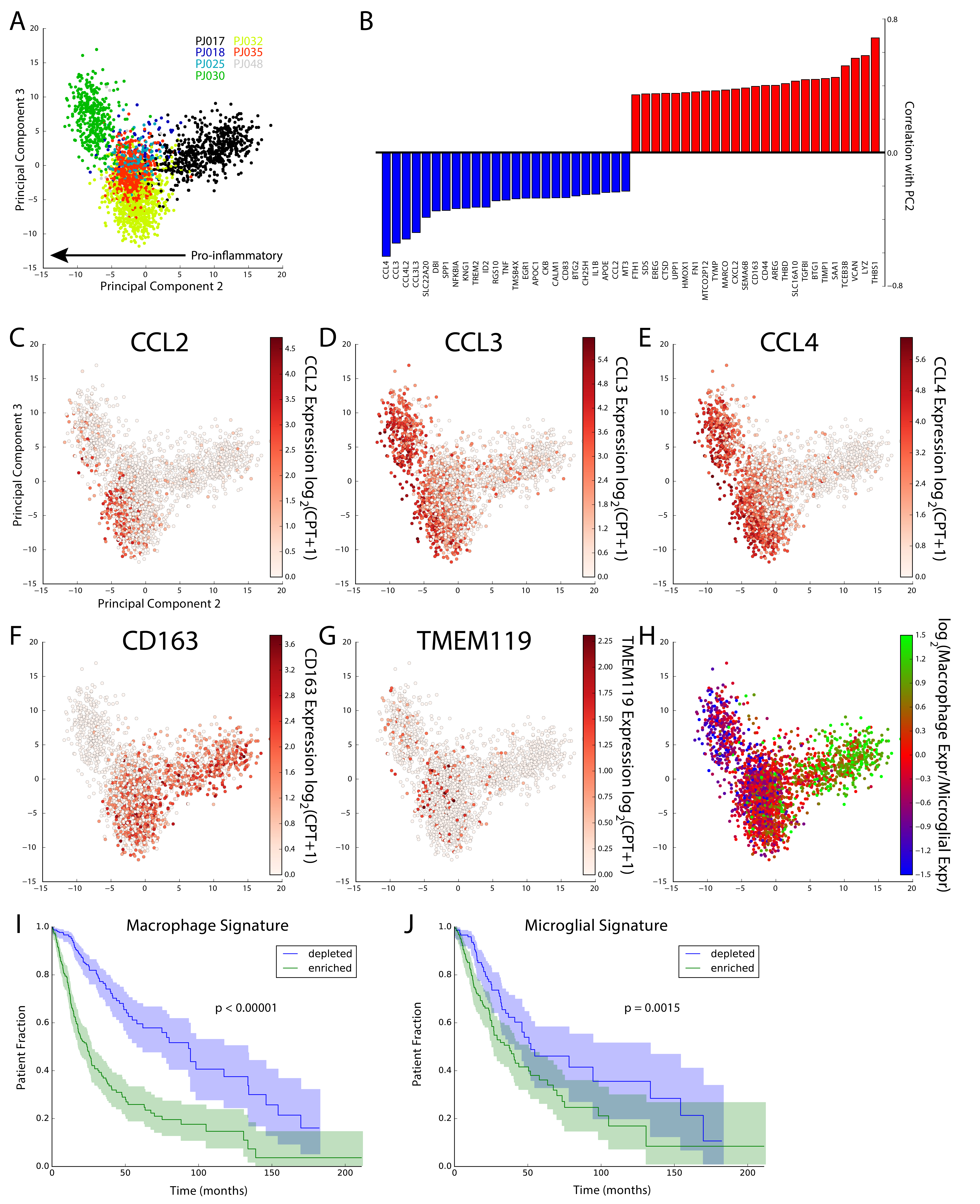
 **Figure S17.** Analysis of myeloid heterogeneity showing microglial and macrophage lineage resemblance, similar to previous reports, and cellular diversity defined by expression of pro-inflammatory cytokines. A) PCA projections of scRNA-Seq profiles from all cells in *CD14*-associated subpopulations (myeloid cells) across the data set, colored by sample-of-origin. B) The second PC is strongly anti-correlated with expression of pro-inflammatory cytokines and correlated with macrophage markers. C)-G) Same as A) but colored with expression of *CCL2*, *CCL3*, *CCL4*, *CD163*, and *TMEM119*, respectively. H) Same as A) but colored by the log-ratio of the expression of microglial vs. macrophage genes as determined from murine lineage tracing studies([14](#_ENREF_14)).

**
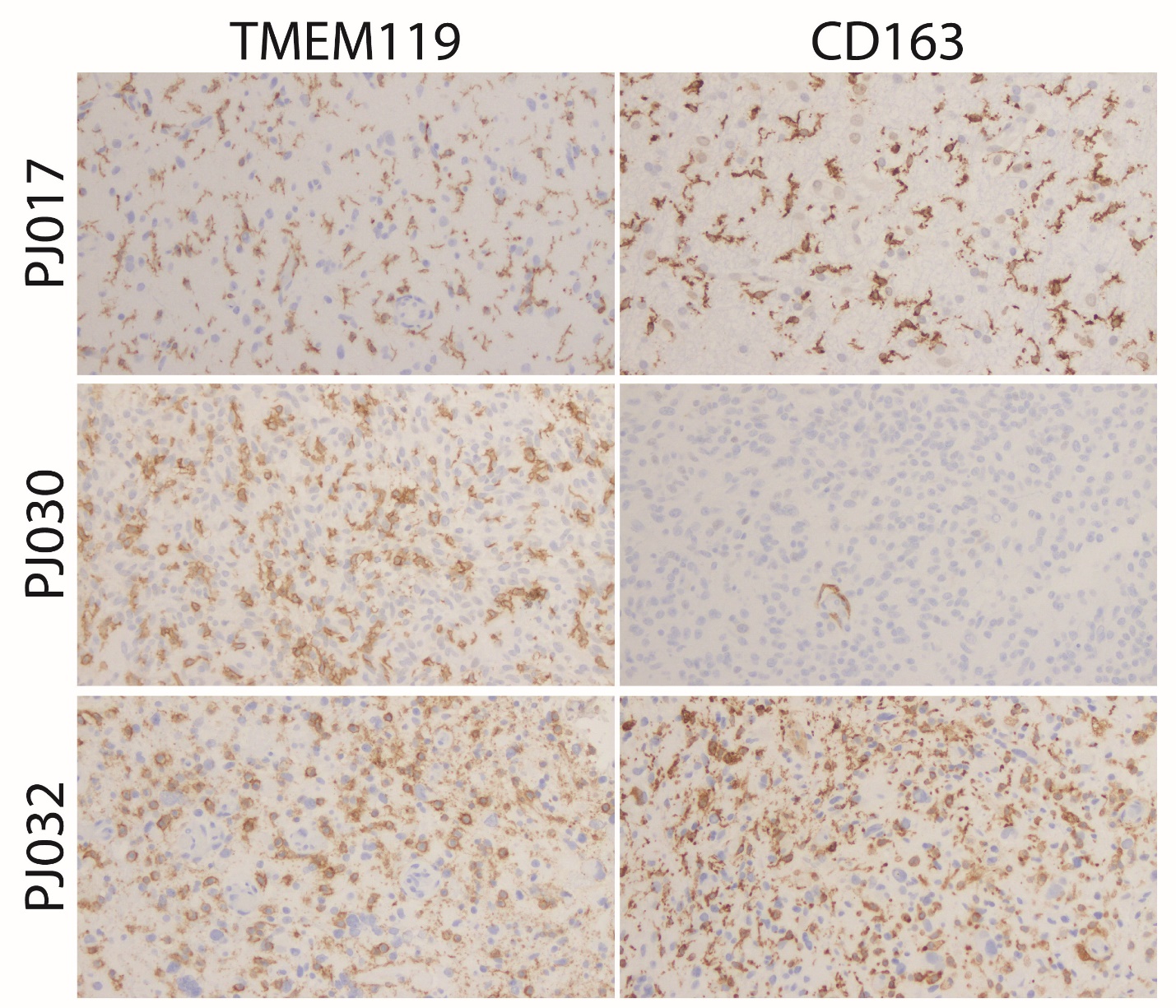
**

**Figure S18** Immunohistochemical staining of TMEM119 and CD163 in three different tumor specimens. PJ017 and PJ032 both show a large number of cells with high levels of TMEM119 and CD163. However, while PJ030 has many cells that stain positive for TMEM119, there are very few CD163+ cells. These results are consistent with the scRNA-Seq data for these three tumors shown in **Figure S15**.

| **Sample** | **sex** | **location** | **Diagnosis** | **IDH1 Status** | **EGFR Status** | **Cells Profiled** | **Subtype** |
| --- | --- | --- | --- | --- | --- | --- | --- |
| PJ016 | F | right frontal | Glioblastoma, WHO grade IV | R132H | not amplified | 3,085 | Proneural |
| PJ017 | M | left temporal | Glioblastoma, WHO grade IV | wt | amplified | 1,261 | Mesenchymal |
| PJ018 | M | left temporal | Glioblastoma, WHO grade IV | wt | not amplified | 2,197 | Proneural |
| PJ025 | M | right frontal | Glioblastoma, WHO grade IV | wt | amplified | 5,924 | Classical |
| PJ030 | F | left temporal | Anaplastic Astrocytoma, WHO grade III | wt | not amplified | 3,097 | Classical |
| PJ032 | F | left temporal | Glioblastoma, recurrent | wt | amplified in recurrent tumor | 1,387 | Mesenchymal |
| PJ035 | M | left temporal | Glioblastoma, recurrent | wt | amplified in initial resection | 3,768 | Classical |
| PJ048 | M | right parietal | Glioblastoma, WHO Grade IV | wt | not amplified | 3,084 | Proneural |

**Table S1.** Summary of patient and sample data. Two of the samples are post-treatment recurrent GBM. All other samples are from primary tumor sections.

| E2F5 | TYMS |
| --- | --- |
| CCNE1 | CCNA2 |
| CCNE2 | CCNF |
| CDC25A | CENPF |
| CDC45L | TOP2A |
| CDC6 | BIRC5 |
| CDKN3 | BUB1 |
| E2F1 | BUB1B |
| MCM2 | CCNB1 |
| MCM6 | CCNB2 |
| NPAT | CDC2 |
| PCNA | CDC20 |
| SLBP | CDC25B |
| BRCA1 | CDC25C |
| CDKN2C | CDKN2D |
| DHFR | CENPA |
| MSH2 | CKS1 |
| NASP | CKS2 |
| RRM1 | PLK |
| RRM2 | STK15 |

**Table S2.** Cell cycle control gene set.

| SAA3P | RP11-745C15.2 | SOD2 | COL6A2 | TNFAIP2 | MT1L | FOSB | LRIG3 | MT2P1 | MRC2 |
| --- | --- | --- | --- | --- | --- | --- | --- | --- | --- |
| PI3 | HP | CYR61 | SERPING1 | S100A10 | MT1G | TNC | MGP | MT1H | MOXD1 |
| SAA2 | PTX3 | C8orf4 | MGST1 | SLC16A7 | GADD45B | NAMPTP1 | DPYD | USP38 | F3 |
| SAA1 | NNMT | CP | NFKBIZ | FTLP2 | TIMP1 | C6orf141 | MT1HL1 | LGALS3 | CXCL3 |
| PLA2G2A | C1S | EFEMP1 | AC073325.2 | SAA4 | MT1E | DCN | TPD52L1 | SLC39A14 | RDH10 |
| SLPI | CCL2 | SPOCD1 | SNX10 | PDPN | TNFAIP3 | COL6A1 | IL32 | SCG2 | OSMR |
| CFH | C1R | CD44 | MT1XP1 | FAM20C | CXCL14 | CA12 | MT1P3 | JUNB | SERPINE1 |
| FAM177B | CHI3L2 | PTRF | GLIPR1 | ABCC3 | CEBPD | TAGLN | RGS2 | CSF1 | TMEM132E |
| MSMP | NAMPT | AEBP1 | GBP1 | ACTN1 | VCAM1 | GBP2 | HIST2H2AA4 | TAGLN2 | PTGS2 |
| LTF | CHI3L1 | CXCL2 | CHL1 | NPNT | FN1 | YBX3 | MT1A | MAN1C1 | FTLP5 |

**Table S3.** List of genes that are significantly more frequently expressed in the transformed cells from PJ017 and PJ032 than in the remaining tumors (top 100 genes).

**Table S4.** List of genes specific to macrophages or microglia based on murine lineage tracing studies and after removal of genes that are more highly expressed in transformed glioma cells in our data set.

| **Macrophage Gene Set** | **Microglial Gene Set** |
| --- | --- |
| GAB3 | SERPINE1 |
| CCDC69 | NAV3 |
| NR4A3 | SNAP47-AS1 |
| GPR35 | SCN1B |
| PLAC8 | OTUD1 |
| IL1RN | CHST11 |
| CIITA | CD37 |
| PLBD1 | COX6A2 |
| CD74 | MEF2C |
| LILRB3 | ABCB4 |
| TACSTD2 | MLPH |
| FAM107B | FAM72A |
| ADRBK2 | KCNMA1 |
| FOSL2 | TAL1 |
| MS4A7 | RGL3 |
| GPR65 | SLC2A5 |
| CNN2 | PHACTR1 |
| TNFRSF4 | FAM149A |
| IL2RG | OLFML3 |
| CREM | ADRB2 |
| SIGLEC1 | SUSD3 |
| TNFAIP2 | CST7 |
| SYTL3 | RNF128 |
| NR4A2 | CCL4TNF |
| FLT3 | OLFML2B |
| METRNL | KLF2 |
| GYPC | DNAJA4 |
| PTGER2 | GLUL |
| PRDM1 | GPR34 |
| ANXA11 | PDE3B |
| TREML4 | ADORA3 |
| SAMHD1 | CTSD |
| GPR141 | HEXB |
| ST8SIA4 | BIN1 |
| DSE | PDGFB |
| SLAMF7 | SDK1 |
| HTR7 | MLXIPL |
| ANPEP | GRAP |
| RGS1 | COL6A3 |
| SH3BGRL | DDX43 |
| CARD11 | RASGRP3 |
| PIM1 | CD81 |
| NLRC4 | P2RY12 |
| LSP1 | GPR84 |
| MTHFR | LBX2-AS1 |
| IFITM2 | TMEM119 |
| TNIP3 | RHOB |
| CTDSP1 | PLXDC2 |
| QPCT | TLN2 |
| LRRC8C |  |
| APOC4-APOC2 |  |
| KMO |  |
| ADAMDEC1 |  |
| CYTIP |  |
| CD40 |  |
| DPEP2 |  |
| RUNX3 |  |
| ST3GAL1 |  |
| TLR5 |  |
| ALPK1 |  |
| DRAM1 |  |
| TREM1 |  |
| GAPT |  |
| RAB11FIP1 |  |
| CLEC4E |  |
| MAP4K1 |  |
| PLA2G7 |  |
| TIMD4 |  |
| APOC2 |  |
| AIM1 |  |
| CCL22 |  |
| ADAMTSL4 |  |
| CCR2 |  |
| CFP |  |
| PTPN7 |  |
| KCNAB2 |  |
| RBMS1 |  |
| ZC3H12D |  |
| EMB |  |
| PPARG |  |
| RUNX2CD300A |  |
| CLEC12A |  |
| CYTH1 |  |
| MALT1 |  |
| DNASE1L1 |  |
| PLTP |  |
| FFAR2 |  |
| TNFRSF18 |  |
| CYBB |  |
| RGS18 |  |
| TGFBI |  |
| TLR8 |  |
| MNDA |  |
| HIC1 |  |
| CD80 |  |
| DOCK5 |  |
| KYNU |  |
| CCR1 |  |
| MAPK13 |  |
| AOAH |  |
| GALNT6 |  |
| CLEC9A |  |
| PSTPIP1 |  |
| PDE8A |  |
| VDR |  |
| EVI2B |  |
| TFEC |  |
| ZMYND15 |  |
| ARHGAP15 |  |
| DAPK1 |  |
| CRIP1 |  |
| FUT4 |  |
| SYNGR2 |  |
| EMILIN2 |  |
| FXYD5 |  |
| CCL17 |  |
| JAK2 |  |
| ITGB7 |  |
| DOK3 |  |
| NFIL3 |  |
| FGR |  |
| IL7R |  |
| RASGRP4 |  |
| BHLHE40 |  |
| ITGA4 |  |
| IL10 |  |
| PMAIP1 |  |
| THBS1 |  |
| AHR |  |
| HP |  |
| CXCR4 |  |
| PLXND1 |  |
| HGF |  |
| NAPSA |  |
| CXCL3 |  |
| CD93PLXNC1 |  |
| FPR2 |  |
| SPINT2 |  |
| B3GNT8 |  |
| S100A11 |  |
| ATP8B4 |  |
| MYO1G |  |
| NFKBIE |  |
| DCSTAMP |  |
| APOBR |  |
| IL2RA |  |
| RIN3 |  |
| SERPINB9 |  |
| MYOF |  |
| MEFV |  |
| LILRA6 |  |
| SMPDL3A |  |
| AQP9 |  |
| PRKCH |  |
| STOM |  |
| THBD |  |
| SEMA4A |  |
| GPR132 |  |
| GSN |  |
| STAT4 |  |
| CSF2RB |  |
| PTPN22 |  |
| ITGA9 |  |
| CXCL16 |  |
| TBC1D10C |  |
| IL1R2 |  |
| CXCL2 |  |
| SELL |  |
| CCL1 |  |
| AMICA1 |  |
