# Supplementary figures and images for: Single-cell transcriptome analysis of lineage diversity in high-grade glioma
Source: Genome Med. 2018 Jul 24;10:57. doi: 10.1186/s13073-018-0567-9 (PMC6058390; doi:10.1186/s13073-018-0567-9)

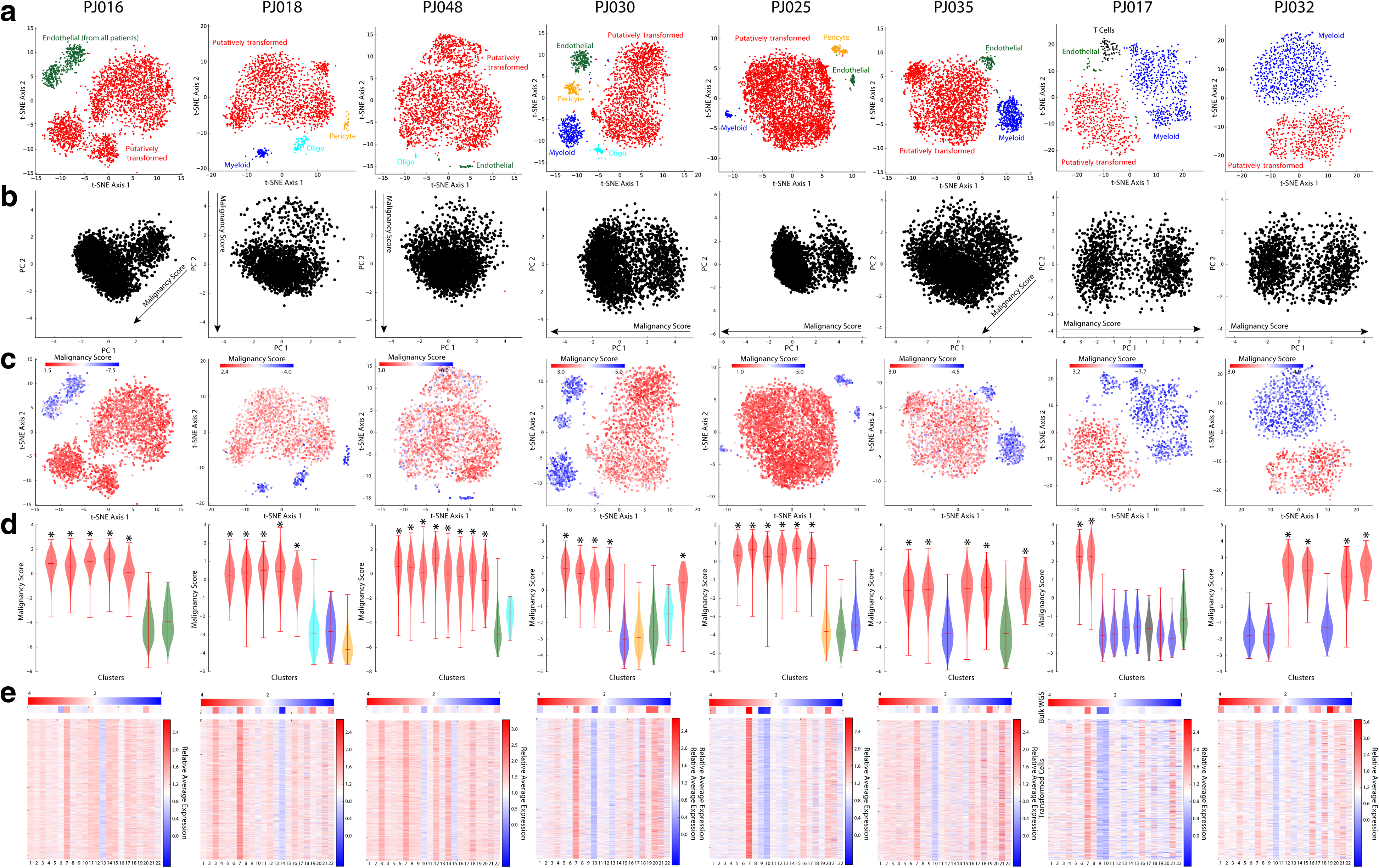

Supplement: Supplementary file 2 — High resolution version of Figure 1. (TIF 3737 kb) [file 13073_2018_567_MOESM2_ESM.tif]
